# Neuroprotective Effects of Hesperidin and CK2 Inhibitor DRB on Aβ_1-42_-Induced Neurotoxicity in Differentiated SH-SY5Y Cells

**DOI:** 10.1007/s12035-025-05082-2

**Published:** 2025-05-30

**Authors:** Hamiyet Eciroglu-Sarban, Pinar Altin-Celik, Pelin Kelicen-Ugur, Hamiyet Donmez-Altuntas

**Affiliations:** 1 Vocational School of Health Services, Alanya Alaaddin Keykubat University, 07425 Antalya, Türkiye; 2 Institute of Health Sciences, Erciyes University, 38030 Kayseri, Türkiye; 3 Department of Medical Biology, Medical Faculty, Erciyes University, 38030 Kayseri, Türkiye; 4 Department of Pharmacology, Faculty of Pharmacy, Hacettepe University, 06410 Ankara, Türkiye

**Keywords:** Alzheimer's disease, CK2, DRB, Hesperidin, Neuronal differentiation, SH-SY5Y neuroblastoma cells

## Abstract

There is still no approved treatment for Alzheimer’s disease (AD), a progressive neurodegenerative disorder characterized by amyloid plaques, neurofibrillary tangles, and synaptic dysfunction. In an in vitro AD model, this study aimed to comparatively assess the neuroprotective effects of the citrus flavonoid Hesperidin and the casein kinase 2 (CK2) inhibitor 5,6-dichloro-1-β-D-ribofuranosyl benzimidazole (DRB) as potential therapeutic targets for AD. First, SH-SY5Y neuroblastoma cells were differentiated into cholinergic neuron-like cells using all*-trans* retinoic acid (RA) and brain-derived neurotrophic factor (BDNF). Then, to generate an in vitro AD model, 20 μM Aβ_1–42_ was applied to induce neurotoxicity in differentiated SH-SY5Y cells. The neuroprotective effects of the CK2 inhibitor DRB and Hesperidin on the in vitro AD model were evaluated using MTT, RT-qPCR, and ELISA methods. Both Hesperidin and DRB, at high concentrations, reduced cell viability in differentiated SH-SY5Y cells for 24 and 48 h (*p* < 0.05 to *p* < 0.01). Pre-treatment with Hesperidin at 25 and 50 µM and DRB at 0.25 and 0.5 µM for 24 h increased ADAM10 gene expression and decreased BACE1 gene expression, both of which are associated with AD markers, compared to the 20 µM Aβ_1-42_ treatment group (*p* < 0.05). Pre-treatment with the DRB at 0.25 and 0.5 µM concentrations for 24 h decreased CK2α gene expression in the in vitro AD model compared to the 20 µM Aβ_1-42_ treatment group (*p* < 0.05), whereas Hesperidin had no effect (*p* > 0.05). Both pre-treatment with Hesperidin and DRB significantly decreased Aβ_1-42_ levels (*p* < 0.01), p-Tau (T181) levels (*p* < 0.05), and the Bax/Bcl-2 ratio (*p* < 0.05). As a result, our study showed that both Hesperidin and DRB inhibited Aβ production by suppressing the amyloidogenic pathway and activating the non-amyloidogenic pathway while also exerting an inhibitory effect on neuronal apoptosis. CK2 may be a potential therapeutic target and could contribute to the pathophysiology of AD. However, these findings should be validated by further studies.

## Introduction

Alzheimer's disease (AD) is a progressive neurodegenerative disorder characterized by cognitive impairment and the loss of functional abilities [1]. According to the World Health Organization (WHO), AD is the most common cause of dementia and represents a growing global health challenge due to the increasing elderly population [2].

Key pathological features of AD include extracellular amyloid plaques, intraneuronal neurofibrillary tangles (NFTs), deterioration of synaptic function, dysfunction in the cholinergic system, and the neuronal atrophy [1, 3]. Amyloid plaques primarily form due to the proteolysis of transmembrane amyloidogenic precursor protein (APP) by β-secretase and γ-secretase in the amyloidogenic pathway, resulting in the accumulation of toxic forms of proteins with high fibrillization potential, such as amyloid beta_1–42_ (Aβ_1–42_) [1, 4, 5]. However, if APP protein is cleaved by α-secretase in the non-amyloidogenic pathway, a soluble form of the protein is produced and is not associated with the disease. Therefore, according to the current data, some changes in the expression of APP proteolysis-related genes–such as the APP, ADAM10 (encoding α-secretase), BACE1 (encoding β-secretase), Presenilin1 (PSEN1), and PSEN2 (genes that regulate the catalytic function of γ-secretase)–contribute significantly to the pathogenesis of the disease [6, 7]. Hyperphosphorylation of tau protein, one of the important pathological findings, triggers the formation of insoluble intraneuronal NFTs. This process disrupts microtubule stabilization, impairs the neuronal transport system, and compromises cellular structure [8, 9]. One of the key views in the progression of AD pathology is that tau and Aβ work in tandem, amplifying the toxic effects of each other [9]. Although there is strong evidence that Aβ induces severe apoptosis in neuronal atrophy and AD, the molecular mechanism of AD has not yet been fully elucidated [5, 10].

The limited number of FDA-approved drugs available for the treatment of AD merely provide symptomatic relief. Therefore, understanding the pathogenesis of AD and developing effective treatment strategies remain critically important [7, 11]. Protein kinases, which are very important in cellular processes, are thought to play critical roles in the brain and many tissues, making them attractive therapeutic targets for AD [12].

Protein kinase 2 (CK2, previously known as casein kinase II) and its substrates are implicated in development, neurogenesis, synaptic plasticity, synaptic transmission, and information storage in the brain [13–15]. CK2 is a heterotetrameric serine/threonine kinase composed of two catalytic α subunits and two regulatory β subunits, with over 300 substrates [15, 16]. Notably, it has been revealed that the human brain’s prefrontal cortex and hippocampus have higher levels of expression for the constitutively active catalytic subunit CK2α [16]. Limited animal studies have demonstrated that CK2 promotes the formation of NFTs in association with AD, with its activity increased in the presence of pathogenic Aβ oligomers, suggesting that elevated CK2 activity contributes AD [14, 17, 18]. Conversely, other perspectives suggest that CK2 activity may reduce Aβ peptide production [19, 20]. These contradictory findings highlight the need for further investigation into CK2’s role in AD pathology [12, 21].

CK2 inhibitors, such as 5,6-dichloro-1-β-D-ribofuranosyl benzimidazole (DRB) and 4,5,6,7-tetrabromo-1H-benzotriazole (TBB), are used to investigate the function of CK2. Moreover, these inhibitors are gaining prominence in research focused on elucidating the processes of neurodegenerative diseases, such as AD and Parkinson’s disease (PD). DRB interacts with both the ATP-binding site and the CK2α site, and is considered to exhibit greater selectivity than CK2 inhibitors, such as 2-dimethyl-amino-4,5,6,7-tetrabromo-1H-benzimidazole (DMAT) and TBB [14, 15, 22, 23]. Alongside current CK2 inhibitors, the impact of newly identified therapeutic drugs will also play a crucial role in this research domain [14, 23].

In recent years, natural bioactive compounds have attracted interest as potential therapeutic agents for the treatment of neurodegenerative diseases. Hesperidin is a flavonoid present in citrus fruits, especially in the peels of oranges and lemons. Some research examining its neuroprotective properties suggests that Hesperidin may positively influence brain health and could help in the prevention or management of neurodegenerative diseases like AD [24, 25]. While Hesperidin has predominantly been assessed in in vivo models of AD, there is limited research available in the literature regarding its effects in in vitro settings [24–27]. However, its impact on CK2 expression in AD has not been previously evaluated.

Given this background, the present study aimed to evaluate and compare the neuroprotective effects of DRB, a selective CK2 inhibitor, and Hesperidin, a citrus flavonoid, on an in vitro AD model established by inducing neurotoxicity with Aβ_1–42_ in differentiated SH-SY5Y cells. This approach may offer novel insights into the molecular interplay between CK2 signaling, APP processing, and potential neuroprotective strategies in AD.

## Materials and Methods

### Preparation of Chemicals

The chemicals used in this study were commercially obtained and prepared according to the manufacturers'instructions. All*-trans* retinoic acid (RA; Bldpharm, China) was prepared as a 10 mM stock solution in dimethyl sulfoxide (DMSO; Sigma-Aldrich, USA) and was immediately diluted with medium before use. The sensitivity of RA to light, heat, and air was taken into account during the preparation and use of the solution. Brain-derived neurotrophic factor (BDNF; Prospec, Israel) stock solution was prepared at 100 µg/ml in sterile 18 M-cm distilled water containing 0.1% bovine serum albumin (Sigma Aldrich, USA) and stored at −80 ^0^C. Hesperidin (Sigma-Aldrich, USA) stock solution was prepared in pyridine (Sigma-Aldrich, USA) at 50 mg/mL and stored at + 4 ^0^C for short-term use. 5,6-Dichlorobenzimidazole 1-β-D-ribofuranoside (DRB; Sigma-Aldrich, USA) was prepared at a concentration of 100 mM in DMSO and stored at −20 ^0^C until use.

### Preparation of Aβ Peptides

Human Aβ_1–42_ lyophilized powder (GenScript, USA) was dissolved in 3% ammonium hydroxide (NH_4_OH; Carlo Erba, Italy) (pH > 9.0). The Aβ_1–42_ stock solution was prepared in 1X phosphate‑buffered saline (PBS, Capricorn, Germany) (pH 7.4) and stored at −80 ^0^C. Prior to use, the Aβ_1–42_ stock solution was incubated at 37 ^0^C for 24 h for the fibrilization and then freshly diluted to the determined concentrations with medium, as described in the literature [28, 29].

### Cell Culture of SH-SY5Y Cells

In this study, the human SH-SY5Y neuroblastoma cell line (Sigma-Aldrich, USA) was used due to its ability to differentiate into neuron-like cells. Cells were cultured in Dulbecco's modified Eagle's medium (DMEM)/Ham's F-12 medium (Capricorn, Germany) supplemented with 10% heat-inactivated fetal bovine serum (FBS, Capricorn, Germany), and 1% (v/v) penicillin/streptomycin (Capricorn, Germany). Cells were incubated at 37 ^0^C, 5% CO_2,_ and 95% humidity. The culture medium was replaced every 2 days, and when the cells reached 80–90% confluence, subculture was performed after trypsinization [30].

### Differentiation of SH-SY5Y Cells

**Fig. 1:**
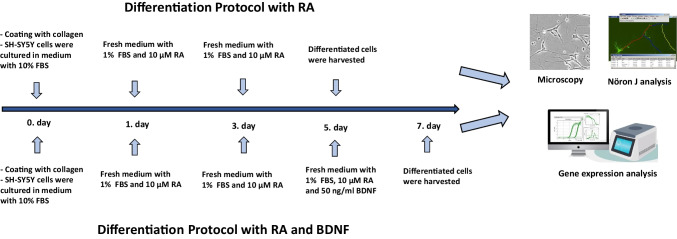
Differentiation of SH-SY5Y cells. SH-SY5Y cells were cultured on collagen-coated plates for the first 24 h in medium containing %10 FBS in both differentiation protocols. In the differentiation protocol with RA, cells were treated with medium containing 1% FBS and 10 µM RA for five days. In the differentiation protocol with RA and BDNF, cells were treated with the first differentiation medium (1% FBS and 10 µM RA) for the first five days, followed by treatment with the second differentiation medium (1% FBS, 10 µM RA, and 50 ng/ml BDNF) for two days

SH-SY5Y cells were differentiated into cholinergic neurons by inducing RA and BDNF. Two distinct protocols for differentiation were applied. First, the 6-well plates were coated with sterile 50 µg/ml type I collagen (Gibco, USA) for approximately 1 h [31]. SH-SY5Y cells were cultured in medium supplemented with 10% FBS and 1% penicillin–streptomycin at a density of 2 × 10^4^ cells/cm^2^ for 24 h. Then, the cells were treated with the first differentiation medium, containing 1% FBS and 10 µM RA, and the medium was refreshed every 2 days for 4 days (differentiation protocol with RA). On the 5 th day, the cells were treated with the second differentiation medium, containing 1% FBS, 10 µM RA, and 50 ng/ml BDNF, for the next 2 days [31, 32]. The differentiation protocol was completed at the end of the 7 th day (differentiation protocol with RA and BDNF), and the differentiated SH-SY5Y (d-SH-SY5Y) cells were used for further experiments (Fig. 1).

To verify the two differentiation protocols, the morphological changes and gene expression levels of neuronal and cholinergic markers (MAP2, NeuN, ChAT, and AChE) during the differentiation of SH-SY5Y neuroblastoma cells into mature, cholinergic neurons were compared between three groups: the Control group (no treatment), the RA group (treated with RA for 5 days), and the RA + BDNF group (treated with RA for 5 days, followed by BDNF treatment for 2 days). Morphological differences between groups in randomly selected areas were visualized using an inverted microscope (Zeiss Axio inverted microscope, Germany) with a 40 × objective. Neurite lengths and numbers were examined using the NeuronJ (ImageJ) program, with data from six randomly selected cells in each field proportioned to the total percentage amount [33–35]. Gene expression levels were evaluated using the quantitative real-time PCR (RT-qPCR) method (see Section 2.7).

### Cell Viability Assessment

Cell viability in the toxicity model and drug treatments was assessed using the MTT assay (3-(4,5-dimethylthiazol-2-yl)−2,5-diphenyltetrazolium bromide; Sigma-Aldrich, USA). At the end of the experimental period, cells were incubated with MTT solution at a final concentration of 0.5 mg/ml for 3 h at 37 ^0^C, under 95% humidity and 5% CO_2_. The medium was then aspirated, and 100 µl of DMSO was added to each well, followed by shaking until the formazon crystals were dissolved. The intensity of the resulting color in the plate was measured spectrophotometrically at 630 nm and 570 nm absorbance on the ELISA reader (Synergy H1, Biotek, USA). The experiments were performed with at least three (or four) replicates. The results were evaluated by calculating the percentage ratio compared to the control [36].

### In Vitro Modeling of AD and Hesperidin and DRB Treatments on d-SH-SY5Y Cells

SH-SY5Y cells were cultured in 96-well plates pre-coated with collagen (50 µg/ml) (5 × 10^3^ cell/well). The differentiation protocol with RA and BDNF was applied as specified in Section 2.3. First, to assess the cytotoxic effects of Hesperidin and DRB on d-SH-SY5Y cell viability, the d-SH-SY5Y cells were treated at different concentrations of Hesperidin (6.25, 12.5, 25, 50, 75, 100, and 200 µM) and DRB (0.25, 0.5, 1, 3.9, 7.8, 15.62, 31.25, 62.5, 125, and 250 µM) for 24 and 48 h and evaluated using the MTT assay. Additionally, the effects of 0.1% Pyridine (solvent for Hesperidin) and 0.1% DMSO (solvent for DRB) on cell viability were also examined. Then, d-SH-SY5Y cells were treated with increasing concentrations (2.5, 5, 10, 20, 40, and 50 µM) of Aβ_1–42_ for 24 and 48 h to determine the toxic concentration, the IC_50_ value, using the MTT assay. In subsequent experiments, d-SH-SY5Y cells treated with 20 μM Aβ_1–42_ for 24 h were used to generate an in vitro AD model.

To assess the neuroprotective effect of Hesperidin and DRB, the treatment groups were pretreated with different concentrations of Hesperidin (6.25, 12.5, 25, 50, 75, 100, and 200 µM) and DRB (0.25, 0.5, 1, 3.9, 7.8, 15.62, 31.25, and 62.5 μM) for 24 and 48 h. Since DRB concentrations of 125 and 250 µM showed high cytotoxicity, further studies were conducted at lower concentrations. Subsequently, the medium was removed, and the medium with 20 μM Aβ_1–42_ was added to the cells for 24 h.

### Gene Expression Analysis by RT-qPCR

Gene expression levels of MAP2, NeuN, ChAT, and AChE (neuronal and cholinergic markers) in the differentiation of SH-SY5Y neuroblastoma cells into mature and cholinergic neurons, and of ADAM10, BACE1, and CK2α for evaluating the neuroprotective effects of the Hesperidin and DRB in the in vitro AD model were assessed by RT-qPCR. Gene expression levels were evaluated in the control group (d-SH-SY5Y cells), Aβ_1–42_ group (20 µM), Hesperidin (25 and 50 µM) groups, and DRB (0.25 and 0.5 µM) groups.

Total RNA was isolated from the experimental groups using the Total RNA Purification Isolation Kit (Jena Bioscience, Germany). The purity of the extracted RNA was assessed using the Multimode Microplate Reader (Synergy H1, Biotek, USA) by measuring the absorbance ratios A260/A280 (1.8–2.1). Subsequently, cDNA synthesis was carried out from total RNA using the cDNA synthesis kit (A.B.T. Laboratory Industry, Ankara, Türkiye). Before use, RNA samples were stored at −80 ^0^C and cDNA at −20 ^0^C.

RT-qPCR reactions were performed on a Roche LightCycler 96 Instrument, using the RT2 SYBR Green qPCR Master Mix Kit (A.B.T. Laboratory Industry, Ankara, Türkiye). The glyceraldehyde-3-phosphate dehydrogenase (GAPDH) gene was used as an internal control. The thermal protocol for RT-qPCR was as follows: pre-incubation at 95 °C for 5 min, followed by 45 cycles of 15 s at 95 °C, 30 s at 60 °C, and 30 s at 72 °C. Primer sequences are presented in Table 1. Relative changes in gene expression were calculated using the cycle threshold (Ct) method according to the 2^−ΔΔCt^ formula, and the data were presented as the fold change in mRNA expression of target genes [37].

**Table 1:** Sequences of forward and reverse primers used in the analysis of gene expression

Gene Symbol	Forward Primer Sequences	Reverse Primer Sequences
MAP2	CTCAGCACCGCTAACAGAGG	CATTGGCGCTTCGGACAAG
NeuN; RBFOX3 F	TTCTCCTTTTCTATTCCCGTTGT	TGTTAGTTTGAATGGTCACACCT
ACHE	AGCAGTACGTTAGTCTGGACCT	TGCTTGCTGTAGTGGTCGAA
ChAT	CAGCCCTGCCGTGATCTTT	TGTAGCTGAGTACACCAGAGATG
ADAM10	TCATGGTGAAACGCATAAGAATCA	AAGACATAGGCCAAACAGTAGTCAT
BACE1	TAACTTTGCAGTGGGTGCTG	GTTGGAGCCGTTGATGAAGA
CSNK2 A1; CK2	GGTGGAATGGGGAAATCAAGAT	GCCATCACGCCACAGTTTC
GAPDH	ACAACTTTGGTATCGTGGAAGG	GCCATCACGCCACAGTTTC

### Enzyme-Linked Immunosorbent Assay (ELISA)

The levels of Aβ_1–42_ (BT Lab, Shanghai, China) and phospho-Tau (p-tau181) (FineTest, Hubei, China), related to AD pathology, and the levels of Bax (BT Lab, Shanghai, China) and Bcl-2 (BT Lab, Shanghai, China), related to the apoptosis mechanism, were measured using sandwich ELISA kits according to the manufacturer's instructions. Absorbance of the supernatants from the experimental groups was measured at 450 nm using the Multimode Microplate Reader (Synergy H1, Biotek, USA).

### Statistical Analysis

All data were presented as mean ± SEM from three (or four) independent experiments. IC_50_ values for Aβ_1–42_, Hesperidin, and DRB were calculated using GraphPad Prism 9.0 software (GraphPad Software, Inc., San Diego, CA). Other statistical analyses were performed using the IBM SPSS software package (version 21.0). Statistical differences between two groups were assessed using one-way analysis of variance (ANOVA) (parametric) or the Mann–Whitney U test (non-parametric). Multiple intergroup comparisons were performed using ANOVA, followed by post-hoc Tukey's HSD, Kruskal–Wallis, or Dunnett's tests. A *p-value of* < 0.05 was considered statistically significant (*p < 0.05, **p < 0.01, ***p < 0.005, ****p < 0.001).

## Results

### Morphological Changes and Neurite Analysis in SH-SY5Y Cell Differentiation

**Fig. 2:**
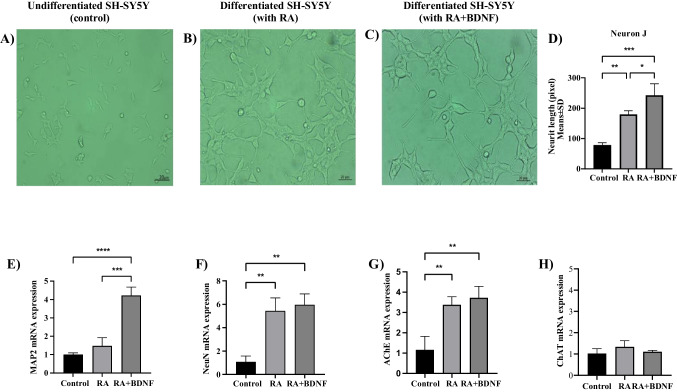
Morphological structure of SH-SY5Y cells and gene expression levels of neuronal and cholinergic neuron markers in two distinct differentiation protocols with RA and RA + BDNF of SH-SY5Y cells. **A** Undifferentiated SH-SY5Y cells (control group with no treatment). **B** RA group (treated with RA for 5 days). **C** RA + BDNF group (treated with RA for 5 days + with BDNF for 2 days). **D** Neurite lengths of SH-SY5Y cells in control, RA, and RA + BDNF groups (Neuron J). **E** and (**F**) Gene expression of MAP2 and NeuN as neuronal markers. **G** and (**H**) Gene expression of AChE and ChAT as cholinergic neuron markers. Results presented as mean ± SEM (Zeiss Axio inverted microscope, Germany; 40 × objective). Significant differences compared to control group: *p < 0.05, **p < 0.01, ***p < 0.005 and ****p < 0.001

Two distinct differentiation protocols were used to promote neuronal differentiation in SH-SY5Y cells: the differentiation protocol with RA and the differentiation protocol with RA and BDNF (Fig. 1). The RA group (treated with RA for 5 days) increased neurite lengths and branching compared to undifferentiated SH-SY5Y cells (control group with no treatment) (*p* < 0.01, Fig. 2B and D). The RA + BDNF group (treated with RA for 5 days + with BDNF for 2 days) also significantly increased neurite lengths and branching, acquiring a neuronal appearance (*p* < 0.001, Fig. 2C and D). Neurite lengths were measured on the 5 th and 7 th days. In addition, a comparison between RA and RA + BDNF differentiation protocols showed that neurite lengths were even longer in the RA + BDNF group (*p* < 0.05, Fig. 2D).

### Gene Expression Analysis of Neuronal and Cholinergic Markers in Differentiated SH-SY5Y Cells

MAP2 and Neun gene expression levels, as neuronal markers, and ChAT and AChE gene expression levels, as markers of cholinergic neurons, were evaluated in differentiated cells. Both the RA group (treated with RA for 5 days) and the RA + BDNF group (treated with RA for 5 days followed by BDNF for 2 days) showed significantly increased MAP2, Neun, and AChE gene expression levels compared to undifferentiated SH-SY5Y cells (control group with no treatment) (*p* < 0.05—*p* < 0.001) (Fig. 2E, F, and G). The RA + BDNF group also exhibited significantly higher levels of MAP2 gene expression in neuronal markers compared to the RA group (*p* < 0.005). Therefore, in subsequent experiments, the RA + BDNF treatment protocol, which best reflects cholinergic neuron transformation, was used for the in vitro AD model.

### Effects of Hesperidin and DRB on Cell Viability in d-SH-SY5Y Cells

**Fig. 3:**
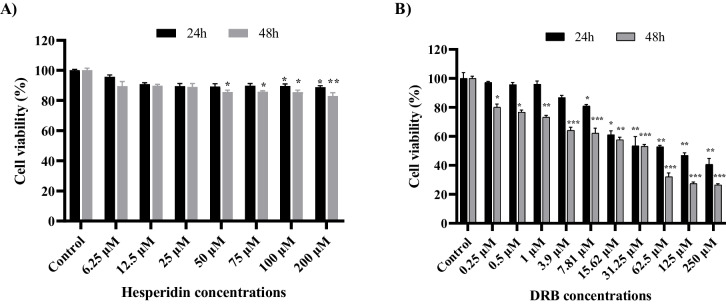
The cytotoxic effects of Hesperidin and DRB on d-SH-SY5Y cells. **A** Effects of Hesperidin on cell viability of d-SH-SY5Y cells for 24 and 48 h. **B** Effects of DRB on cell viability of d-SH-SY5Y cells for 24 and 48 h. Results presented as mean ± SEM. Significant differences compared to control group: *p < 0.05, **p < 0.01 and ***p < 0.005

The cytotoxic effects of Hesperidin and DRB on d-SHSY5Y cells, treated at a series of concentrations for 24 and 48 h, were assessed using the MTT assay (Fig. 3). Hesperidin treatment for 24 and 48 h did not reduce d-SHSY5Y cell viability below 50%. At 48 h, 83.05% cell viability was observed, even at the maximum concentration of Hesperidin at 200 μM (Fig. 3A). Therefore, the IC_50_ value of Hesperidin could not be determined. DRB significantly decreased cell viability at concentrations of 15.62 µM and above for 24 and 48 h in all groups compared to the differentiated SH-SY5Y cells (control group with no Hesperidin or DRB treatment) (*p* < 0.05—*p* < 0.005). The IC_50_ value of DRB was determined as 11.83 μM for 24 h and 5.43 μM for 48 h.

### Protective Effects of Hesperidin and DRB Treatment on d-SH-SY5Y Cell Viability Against Toxicity of Aβ_1–42_

**Fig. 4:**
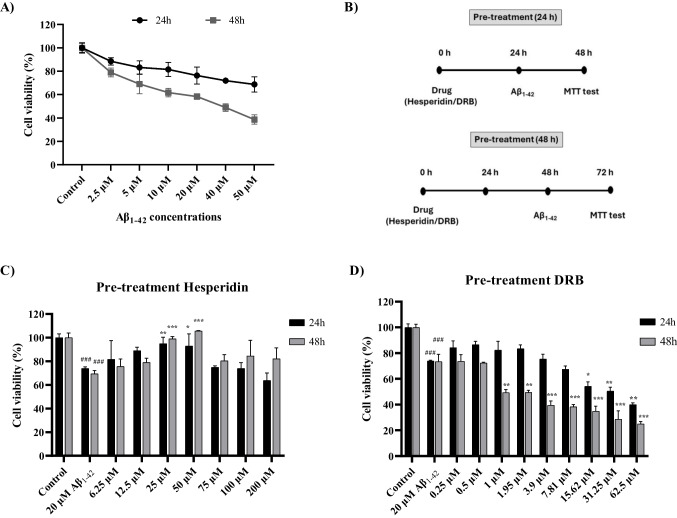
The concentration- and time-dependent effects of Aβ_1–42_ toxicity and Hesperidin and DRB on d-SHSY5Y cell viability, as assessed by the MTT test. **A** Concentration- and time-dependent cytotoxic effects of Aβ_1–42_ on d-SH SY5Y cell-viability. **B** Pre-treatment protocol on d-SHSY5Y cells with Hesperidin and DRB for 24 and 48 h. **C** Protective effect of Hesperidin pre-treatment against Aβ_1–42_ toxicity in d-SHSY5Y cells. **D** Protective effect of DRB pre-treatment against Aβ_1–42_ toxicity in d-SHSY5Y cells. Results presented as mean ± SEM. Significant differences compared to the AB_1–42_ toxicity group: *p < 0.05, **p < 0.01 and ***p < 0.005. Significant difference compared to the control group: ^###^ *p* < 0.005

The effect of increasing concentrations (2.5–50 μM) of the Alzheimer's neurotoxin Aβ_1–42_ on cell viability in d-SHSY5Y cells for 24 and 48 h was determined by MTT assay. Aβ_1–42_ exposure led to a significant decrease in the viability of d-SH SY5Y cells, which was both concentration- and time-dependent (Fig. 4A). Based on the literature [38, 39] and our findings (76.38% viability), we chose to use Aβ_1–42_ at a concentration of 20 µM for 24 h in neurotoxicity (in vitro AD model) experiments.

Hesperidin and DRB were tested for their ability to protect d-SH-SY5Y cells against Aβ_1–42_ toxicity. Cell viability was assessed using the MTT assay after 24 and 48 h of pre-treatment (Fig. 4B). Pre-treatment with Hesperidin (6.25–200 μM) for 24 and 48 h significantly increased the viability of d-SH-SY5Y cells against 20 μM Aβ_1–42_ toxicity, especially at concentrations of 25 and 50 μM (*p* < 0.05 – *p* < 0.005). However, cell viability decreased at concentrations above 75 μM (Fig. 4C).

However, pre-treatment with DRB (0.25–62.5 μM) for 24 and 48 h did not increase cell viability against 20 μM Aβ_1–42_ toxicity. Although not statistically significant, a slight increase in cell viability was observed at concentrations of 0.25 and 0.5 μM for 24 h compared to the Aβ_1–42_ toxicity group (Fig. 4D).

### Pre-Treatment with Hesperidin and DRB Affected Alzheimer's Markers in the In Vitro AD Model

d-SHSY5Y cells were pre-treated with Hesperidin (25 and 50 µM) and DRB (0.25 and 0.5 µM) for 24 h before treatment with 20 μM Aβ_1–42_ for 24 h. To assess the neuroprotective effects of Hesperidin and DRB in the in vitro AD model induced by Aβ_1–42_, we measured the mRNA expression levels of ADAM10 (α-secretase) and BACE1 (β-secretase) genes using RT-qPCR, as well as the Aβ_1–42_ and phospho-Tau (T181) protein levels using ELISA.

**Fig. 5:**
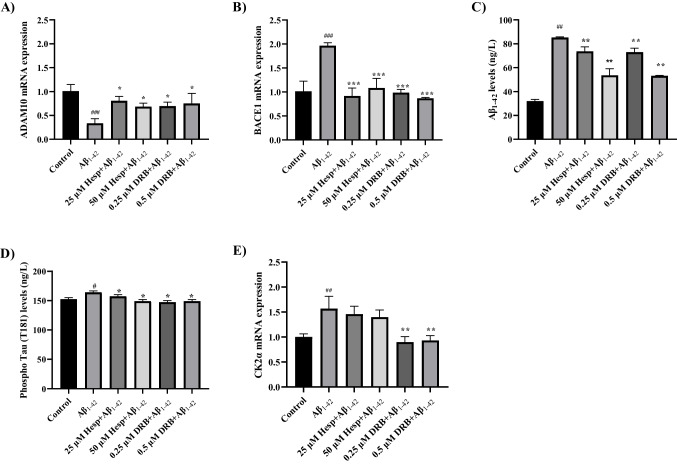
The effects of pre-treatment with Hesperidin (25 µM and 50 µM) and DRB (0.25 µM and 0.5 µM) on Alzheimer's disease markers in the in vitro AD model. **A-B** The effects of pre-treatment with DRB and Hesperidin on ADAM10 and BACE1 mRNA expressions (fold change) in the in vitro AD model (d-SH-SY5Y cells treated with 20 μM Aβ_1–42_ for 24 h). **C-D** The effects of pre-treatment with DRB and Hesperidin on Aβ_1–42_ and phospho-Tau (T181) levels in the in vitro AD model (d-SH-SY5Y cells treated with 20 μM Aβ_1–42_ for 24 h). **E** The effects of pre-treatment with Hesperidin and DRB on CK2α mRNA expressions in the in vitro AD model (d-SH-SY5Y cells treated with 20 μM Aβ_1–42_ for 24 h). Results presented as mean ± SEM. Significant differences compared to Aβ_1–42_ toxicity group: *p < 0.05, **p < 0.01, and ***p < 0.005. Significant differences compared to the control group: ^#^*p* < 0.05, ^##^*p* < 0.01, and ^###^*p* < 0.005

The ADAM10 mRNA expression level was downregulated, while the BACE1 mRNA expression level was significantly upregulated in the Aβ_1–42_ group compared to the control group. The fold changes in ADAM10 and BACE1 gene expressions were 2.9-fold and 1.9-fold, respectively (*p* < 0.005) (Fig. 5A and B). However, Hesperidin at concentrations of 25 µM (2.3-fold) and 50 µM (twofold), as well as DRB at concentrations of 0.25 µM (twofold) and 0.5 µM (2.2-fold), significantly increased ADAM10 mRNA expression levels compared to the Aβ_1–42_ group (*p* < 0.05), while Hesperidin and DRB significantly decreased BACE1 gene expression levels (2.2-fold, 1.8-fold, twofold, and 2.3-fold, respectively) in these groups (*p* < 0.005) (Fig. 5A and B). Hesperidin and DRB had a greater effect on BACE1 gene expression compared to ADAM10 in the in vitro AD model.

Levels of Aβ_1–42_, which are important markers of AD, increased significantly in the Aβ_1–42_ group compared to the control group (*p* < 0.01). However, pre-treatment with Hesperidin (25 and 50 µM) and DRB (0.25 and 0.5 µM) for 24 h significantly decreased Aβ_1–42_ levels compared to the Aβ_1–42_ group (*p* < 0.01) (Fig. 5C). Similarly, phospho-Tau (T181) levels increased in the Aβ_1–42_ group compared to the control group (*p* < 0.05), while pre-treatment with Hesperidin (25 and 50 µM) and DRB (0.25 and 0.5 µM) for 24 h significantly decreased phospho-Tau (T181) levels compared to the Aβ_1–42_ group (*p* < 0.05) (Fig. 5D).

### Pre-Treatment with Hesperidin and DRB Decreased CK2 Gene Expression in the In Vitro AD Model

To investigate the role of CK2 in the in vitro AD model induced by Aβ_1–42_, we measured CK2 mRNA expression in the Aβ_1–42_, Hesperidin and DRB groups using RT-qPCR. CK2 gene expression level was significantly upregulated (1.6-fold) in the Aβ_1–42_ group compared to the control group (*p* < 0.01) (Fig. 5E). Pre-treatment with DRB (0.25 and 0.5 µM), one of the CK2 inhibitors, significantly decreased the CK2 mRNA expression levels compared to the Aβ_1–42_ group (*p* < 0.01). However, Hesperidin pre-treatment (25 and 50 µM) did not cause any significant decrease in CK2 mRNA expression levels compared to the Aβ_1–42_ group in in vitro AD model (*p* > 0.05) (Fig. 5E).

### Pre-Treatment with Hesperidin and DRB Decreased Apoptosis in the In Vitro AD Model

To evaluate the apoptotic effects of pre-treatment with Hesperidin and DRB in the in vitro AD model, pro-apoptotic Bax and anti-apoptotic Bcl-2 protein levels were measured using the ELISA method. The apoptotic index was then determined by calculating the Bax/Bcl-2 ratio.

**Fig. 6:**
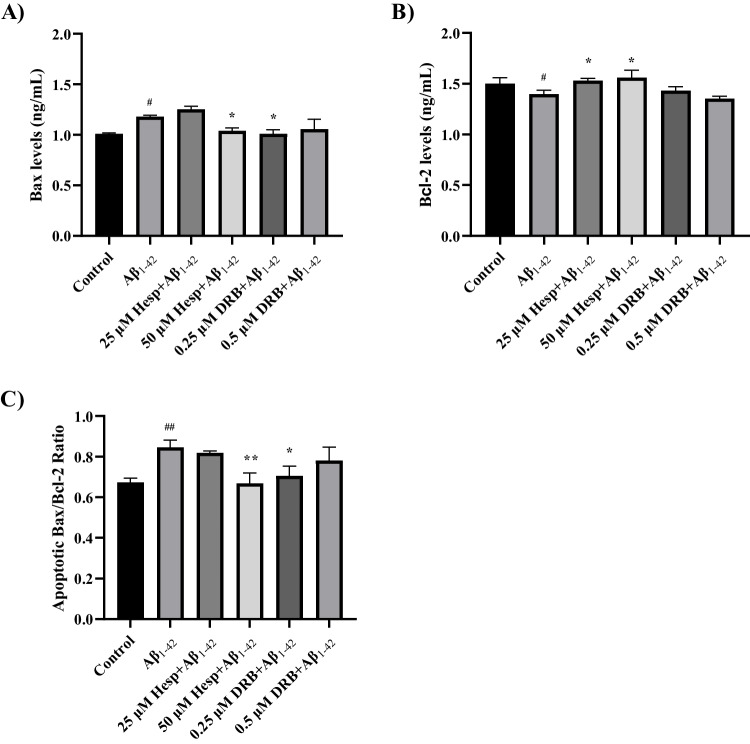
The effects of pre-treatment with Hesperidin and DRB on Bax and Bcl-2 protein levels in the in vitro AD model (d-SH-SY5Y cells treated with 20 μM Aβ_1–42_ for 24 h). **A** Bax protein levels. **B** Bcl-2 protein levels. **C** The apoptotic Bax/Bcl-2 ratio. Results presented as mean ± SEM. Significant differences compared to control group: ^#^* p* < 0.05 and ^##^* p* < 0.01. Significant differences compared to the Aβ_1–42_ toxicity group: * p < 0.05 and ** p < 0.01

The Aβ_1–42_ group showed significantly higher levels of Bax protein compared to the control group, while Bcl-2 protein levels were significantly lower (*p* < 0.05) (Fig. 6A and B). Pre-treatment with 25 and 50 μM Hesperidin enhanced Bcl-2 levels, while pre-treatment with 50 μM Hesperidin reduced Bax levels compared to the Aβ_1–42_ group (*p* < 0.05) (Fig. 6A and B). Pre-treatment with 2.25 μM DRB significantly lowered Bax levels (*p* < 0.05), but Bcl-2 levels did not increase statistically significantly in response to DRB pre-treatment (*p* > 0.05) (Fig. 6A and B). Furthermore, there was a substantial increase in apoptotic index, or the Bax/Bcl-2 protein ratio, between the Aβ_1–42_ group and the control group (*p* < 0.01) (Fig. 6C). However, pre-treatment with 50 μM Hesperidin and 0.25 μM DRB reduced apoptosis by lowering the Bax/Bcl-2 ratio compared to the Aβ_1–42_ group (*p* < 0.01 and *p* < 0.05, respectively) in the in vitro AD model (Fig. 6C).

## Discussion

There is currently no effective treatment for Alzheimer's disease, and its molecular mechanisms have not yet been fully elucidated. To advance our understanding of the condition and facilitate the development of viable therapies, the use of both in vitro and in vivo AD models are essential [1, 7, 40]. In this study, SH-SY5Y cells were treated with sequential RA and BDNF to generate neuron-like differentiated cells (d-SH-SY5Y). Neuronal differentiation was confirmed by both morphological changes and gene expression levels of neuronal markers (MAP2 and Neun) and cholinergic neuron markers (ChAT and AChE). The present study assessed the neuroprotective effects of DRB (a CK2 inhibitor) and Hesperidin in the in vitro AD model using the MTT assay for cell viability, RT-qPCR analysis for gene expression levels of model markers (CK2α, ADAM10, and BACE1), and ELISA for Aβ1–42, phospho-Tau (T181), and apoptotic (Bax and Bcl-2) proteins.

SH-SY5Y cells are among the most commonly preferred cell lines for creating in vitro models in studies related to neurodegenerative diseases due to their ability to differentiate into neuronal cells [31, 41, 42]. Previous studies have shown that SH-SY5Y cells can undergo neuronal differentiation for an Alzheimer's models using various protocols, including treatment with RA [43], sequential treatment with RA and BDNF [31, 32], or in combination with other factors such as NGF, vitamin D, dibutyryl cyclic AMP (cAMP), GlutaMAX, and neurobasal medium [44, 45]. It has been reported that RA and BDNF treatments induce morphological changes in SH-SY5Y cells and increase neurite lengths [31, 32]. Additionally, changes in gene expression levels of MAP2, NeuN, β-tubulin III, AChE, and ChAT have been shown to confirm cholinergic neuron differentiation [31, 32, 46]. Consistent with previous studies, we sequentially treated SH-SY5Y cells with RA for 5 days and BDNF for 2 days (totaling 7 days) to show cholinergic neuron differentiation. The RA + BDNF differentiation protocol resulted in differentiated SH-SY5Y (d-SH-SY5Y) cells with neuron-like morphology, elongated neurites, and significantly higher levels of MAP2, NeuN, and AChE gene expression. We then used the d-SH-SY5Y cells to generate an in vitro AD model.

Aβ_1–42_ concentrations ranging from 10 to 25 µM have frequently been used in prior in vitro studies to create an AD model [28, 47, 48]. The present study demonstrated that Aβ_1–42_ treatment of d-SH-SY5Y cells reduced cell viability in a concentration- and time-dependent manner, providing direct evidence of the cellular toxicity induced by Aβ_1–42_. Numerous studies have revealed that Aβ_1–42_ activates the amyloidogenic pathway, leading to mitochondrial damage and DNA breaks, and ultimately causing cellular and genomic dysfunction [49, 50]. Our results demonstrated that in the toxicity group induced by 20 µM Aβ_1–42_, BACE1 (β-secretase) gene expression was increased, while ADAM10 (α-secretase) gene expression was suppressed. Furthermore, we observed that the Aβ_1–42_ toxicity group exhibited elevated levels of Aβ_1–42_ and phospho-Tau (T181) protein, along with induced apoptosis. Therefore, our findings align with the production of amyloid plaques through the involvement of β- and γ-secretase in the amyloidogenic pathway in AD and are also consistent with those reported in other in vitro AD model studies [6, 48, 50, 51].

Citrus flavonoids, such as Hesperidin, are gaining prominence in the treatment of neurodegenerative diseases due to their antioxidant and anti-inflammatory properties, as well as their ability to cross the blood–brain barrier. Furthermore, they provide a significant therapeutic advantage due to their minimal side effects and low cytotoxicity in healthy cells [24, 25]. Previous studies have shown that Hesperidin exhibits low toxicity in SH-SY5Y cells, even at high concentrations [52, 53], indicating a broad therapeutic safety margin. In SH-SY5Y cells, it has been found to show a protective effect by increasing cell viability against neurotoxic agents such as H_2_O_2_, 6-OHDA, and Bupivacaine [53–55]. In a study on the in vitro AD model, Kuşi et al. showed the protective effect of 5 µM Hesperidin against Aβ_25–35_ [27]. In the present study, we used 20 µM Aβ_1–42_ in d-SH-SY5Y cells to construct an in vitro AD model and examined the protective effects of both Hesperidin and DRB. Our results show that Hesperidin significantly increased cell survival at 25 and 50 µM concentrations, protecting d-SH-SY5Y cells against Aβ_1–42_ neurotoxicity in an in vitro AD model. According to the study by Kuşi et al. [27], besides the fact that it was conducted on different SK-N-AS human neuroblastoma cells, Aβ_1–42_ toxicity is likely higher than Aβ_25–35_ toxicity, which explains why the Hesperidin concentrations in our study were higher than those used in theirs.

On the other hand, Hesperidin has been shown in numerous in vivo models to effectively prevent Aβ accumulation and neuroinflammation, as well as alleviate cognitive and behavioral disorders, although there are limited studies on its role in in vitro AD models [56–59]. Mandour et al. found that Hesperidin reduces memory loss and suppresses Aβ−42, p-Tau, and AChE expressions in AD rats, thereby mitigating the AD-like state [58]. Additionally, Hesperidin has been demonstrated to protect against oxidative stress, apoptosis, and cognitive impairment by reducing acetylcholinesterase activity and the expression of markers related to Aβ biosynthesis [56, 57]. In an in vitro study evaluating the effects of Hesperidin on the immunoreactivity of tau, β-amyloid, and α-synuclein, it demonstrated neuroprotective effect by significantly lowering α-synuclein values in an in vitro AD model produced by Aβ_25–35_ [27]. We also showed that in an in vitro AD model produced by Aβ_1–42_, Hesperidin pre-treatment significantly increased ADAM10 gene expression, decreased BACE1 gene expression, and consequently reduced Aβ_1–42_ peptide and phospho-Tau (T181) levels. Our findings indicate that Hesperidin is a promising agent for the treatment of AD, although further extensive investigation is needed.

The role of CK2 in the pathogenesis of AD is quite controversial. Some clinical studies have associated reduced CK2 activity with AD, suggesting it may contribute to Aβ accumulation [20, 21, 60, 61]. On the other hand, some studies have demonstrated that CK2 expression levels are significantly elevated in the brains of both human and AD animal models, particularly in the hippocampus and temporal cortex, and that this elevation is linked to cognitive impairment [12, 18, 62, 63]. These findings suggest that CK2 is a crucial target in the pathogenesis of AD, prompting researchers to focus on experimental models in this area. To our knowledge, no previous study has investigated DRB in an in vitro AD model. In the present study, CK2 gene expression increased in the in vitro AD model, but DRB (CK2 inhibitor) downregulated CK2 gene expression. Although cell viability against Aβ_1–42_ toxicity was slightly increased by 0.25 and 0.5 μM DRB concentrations for 24 h, this effect was not statistically significant. Furthermore, in previous a study on a Parkinson's disease model, 50 μM DRB was used for 30 min in SH-SY5Y cells, where it was shown that DRB reduced alpha-synuclein p-S129 phosphorylation [64]. However, in our study, we used lower effective doses of DRB, as the longer culture duration and the differentiation of SH-SY5Y cells may have sensitized them. Additionally, using the in vitro AD model, we investigated the relationship between AD markers and the amyloidogenic pathway in relation to CK2 inhibition by DRB. Our results demonstrate that CK2 inhibition has a neuroprotective impact in the in vitro AD model, as evidenced by decreased levels of Aβ_1–42_ and phospho-Tau (T181), along with enhanced ADAM10 gene expression and suppressed BACE1 gene expression. Previous experimental studies using CK2 inhibitors have shown that tau phosphorylation, STAT1 phosphorylation, fast axonal transport (FAT), and GSK3 signaling are all associated with the role of CK2 in AD [14, 18, 65, 66]. Additionally, it has been revealed that improperly folded tau and oligomeric Aβ inhibit synaptic transmission in AD models through aberrant activation of GSK3β and CK2 protein kinases, respectively [66, 67]. Thus, our results support these studies and suggest that CK2 may be a viable therapeutic target for AD. Although various CK2 inhibitors have been discovered to date, their clinical potential remains limited due to low target specificity and other associated drawbacks. The identification of natural compounds with CK2 inhibitory activity could offer a promising avenue for the development of more selective therapeutic strategies [22]. Flavonoids such as quercetin and apigenin have been previously investigated as CK2 inhibitors in various studies [16, 22, 68]. However, the effect of Hesperidin on CK2 expression has not been previously evaluated in an in vitro AD model. In this study, we also assessed whether Hesperidin has CK2 inhibitory effects. Our results showed that hesperidin did not alter CK2 gene expression compared to the Aβ toxicity group. However, our study suggests that the neuroprotective effect of Hesperidin in the in vitro AD model–characterized by decreased levels of Aβ_1–42_ and phospho-Tau (T181), enhanced ADAM10 gene expression, and suppressed BACE1 gene expression–is probably mediated by a mechanism other than CK2 inhibition. As mentioned previously, Hesperidin exerts a neuroprotective effect, probably related to the amyloidogenic pathway.

In neurodegenerative diseases such as AD, apoptosis, which is defined as programmed cell death, is a contributing factor to synaptic dysfunction and cognitive loss [69]. In this study, Aβ_1–42_ toxicity showed increased Bax protein levels, decreased Bcl-2 protein levels, and an elevated Bax/Bcl-2 ratio, indicating that Aβ_1–42_ triggers cellular apoptosis in the in vitro AD model. However, pre-treatment with both Hesperidin and DRB not only modulated Alzheimer's disease-related molecules but also decreased Bax and increased Bcl-2 protein levels, as well as reduced the apoptotic Bax/Bcl-2 ratio in the in vitro AD model, indicating a protective effect in apoptotic cell death against to Aβ_1–42_ toxicity. Similarly, it has been demonstrated to suppress caspase-3/−9 activity in the in vitro AD model induced by Aβ_25–35_ [70]. Additionally, previous studies have shown that Hesperidin inhibits apoptosis and neuronal cell death by decreasing Bax and caspase-3 expression, while increasing Bcl-2 expression in an in vivo AD model [57, 71]. The prevention of apoptosis may be one of the key strategies for treating AD, as neuronal atrophy is a significant pathological finding. Alzheimer's patients’ brains have been found to exhibit increased active caspase-3 activity, overexpression of pro-apoptotic Bax, and decreased expression of anti-apoptotic Bcl-2 [72–74]. In this study, the protective effects of Hesperidin and DRB may be due to the modulation of cell death signals through neuroinflammation and the amyloidogenic pathway. Based on these findings, we hypothesize that both Hesperidin and DRB may have therapeutic effects in AD by reducing neuronal cell death.

## Conclusion

In the present study, we contributed to the literature by evaluating the gene expression of CK2, whose role in AD pathogenesis has yet to be fully elucidated. Our results demonstrated that the CK2 inhibitor DRB can effectively modulate the increased CK2α gene expression in the Aβ1–42-induced in vitro AD model. Additionally, DRB exerted a protective effect against the amyloidogenic pathway and apoptosis in the in vitro AD model. These findings suggest that CK2 overexpression may play a role in AD pathogenesis. Hesperidin, a flavonoid, showed notable neuroprotective effects by suppressing the amyloidogenic pathway, inhibiting apoptosis, and activating the non-amyloidogenic pathway in the in vitro AD model. Our results also showed that Hesperidin did not inhibit CK2; however, it likely exerted its protective effects through other pathways in the in vitro AD model. Further studies are needed to investigate the mechanisms underlying the role of CK2 in AD in more detail and to develop CK2 inhibitors with lower cytotoxicity.

## Data Availability

No datasets were generated or analysed during the current study.
